# Sustained Release of Amnion-Derived Cellular Cytokine Solution Facilitates Achilles Tendon Healing in Rats

**Published:** 2014-08-04

**Authors:** Maximilian Kueckelhaus, Justin Philip, Rami A. Kamel, Jose A. Canseco, Florian Hackl, Elizabeth Kiwanuka, Mi J. Kim, Ryan Wilkie, Edward J. Caterson, Johan P. E. Junker, Elof Eriksson

**Affiliations:** ^a^Division of Plastic Surgery, Brigham and Women's Hospital, Harvard Medical School, Boston, Mass; ^b^Laboratory for Tissue Engineering and Regenerative Medicine, Department of Anesthesiology, Brigham and Women's Hospital, Harvard Medical School, Boston, Mass; ^c^Harvard-MIT Division of Health Sciences and Technology, Massachusetts Institute of Technology, Cambridge, Mass; ^d^Department of Ophthalmology, Edward S Harkness Eye Institute, Columbia University College of Physicians and Surgeons, New York, Ny

**Keywords:** Achilles tendon, amnion-derived cytokine solution, material testing, sustained release, tendon healing

## Abstract

**Objective:** In the United States, around 50% of all musculoskeletal injuries are soft tissue injuries including ligaments and tendons. The objective of this study is to assess the role of amnion-derived cellular cytokine solution (ACCS) in carboxy-methyl cellulose (CMC) gel in the healing of Achilles tendon in a rat model, and to examine its effects on mechanical properties and collagen content. **Methods:** Achilles tendons of Sprague-Dawley rats were exposed and transected. The distal and proximal ends were injected with either saline or ACCS in CMC, in a standardized fashion, and then sutured using a Kessler technique. Tendons from both groups were collected at 1, 2, 4, 6, and 8 weeks postoperatively and assessed for material properties. Collagen studies were performed, including collagen content, collagen cross-linking, tendon hydration, and immunohistochemistry. Tendons were also evaluated histologically for cross-sectional area. **Results:** Mechanical testing demonstrated that treatment with ACCS in CMC significantly enhances breaking strength, ultimate tensile strength, yield strength, and Young's modulus in the tendon repair at early time points. In context, collagen content, as well as collagen cross-linking, was also significantly affected by the treatment. **Conclusion:** The application of ACCS in CMC has a positive effect on healing tendons by improving mechanical properties at early time points. Previous studies on onetime application of ACCS (not in CMC) did not show significant improvement on tendon healing at any time point. Therefore, the delivery in a slow release media like CMC seems to be essential for the effects of ACCS demonstrated in this study.

Patient-induced and iatrogenic tendon injuries are common clinical problems.^1^ The estimated incidence of tendinous and ligamentous injuries is 166.6/100,000 per year for males and 52.1/100,000 per year for females.^2^^,^^3^ In the United States, around 50% of all musculoskeletal injuries are soft tissue injuries including ligaments and tendons.^4^ More than 32 million traumatic and repetitive motion injuries to tendons and ligaments are recorded each year,^5^ costing the United States an estimated $30 billion annually.^6^

Tendon is made up of dense regular connective tissue rich in collagen,^7^^,^^8^ is characterized by hypovascularity and hypocellularity,^9^ and often demonstrates poor outcomes after repair. Tendon healing passes through the 3 overlapping phases: inflammation, proliferation, and the remodeling. During the inflammatory phase, macrophages release growth factors, which induce the formation of extracellular matrix and fibroblast proliferation.^10^ During the proliferative phase, epitenon cells start to proliferate about 3 days after tendon injury. There is migration and longitudinal deposition of the fibroblasts in the superficial layers of the tendon.^11^ Neovascularization is evident 7 days after tendon repair with formation of vascular channels extending into the repair site. There is also evidence of increased cellularity in the endotenon while maximal proliferation still occurs in the epitenon.^12^ Degradation of the collagen fibers is accompanied by deposition of newly synthesized fine collagen fibers crossing the repair site.^13^ The remodeling phase of tendon tissue is a slow process aiming at the formation of a functional scar that is believed to begin at the peak of proliferation. Deposited type III collagen is slowly broken down by collagenases and replaced with type I collagen, allowing for greater cross-linking and higher tensile strength. A change from cellular to predominantly fibrous tissue takes place in the first few weeks.^14^^-^16 Diameter and cross-linking of the collagen fibrils often remain inferior after the healing process.^17^

Growth factors play a significant role in mediating the tendon healing process, as in other healing tissues.^18^ Vascular endothelial growth factor, platelet-derived growth factor, and transforming growth factor were proven to have an increased expression following injury and during different phases of tendon repair.^19^^-^21

Amnion-derived cells like stem cells possess the ability of differentiating into different cell lineages and produce cytokine growth factors,^22^^-^24 being beneficial in the healing process in various tissue healing models such as brain injury^25^^,^^26^ and acute wound failure models.^27^

The supernatant of amnion-derived multipotent progenitor (AMP) cells, termed amnion-derived cellular cytokine solution (ACCS), was found to contain physiologic levels of cytokines important to healing processes.^28^ A possible positive effect of ACCS in wound healing was suggested in several prior studies.^28^^-^30 Two percent carboxymethyl cellulose (CMC) gel is suggested to be the superior vehicle system for sustained delivery of growth factors compared to biomatrix and to collagen.^31^

The hypothesis of this study was that tendon repair and the mechanical properties after tendon injury could be improved by intratendinous injection of ACCS formulated in a sustained release system.

## MATERIALS AND METHODS

### Cytokine solution preparation

Amnion-derived cellular cytokine solution (Stemnion Inc, Pittsburgh, Pennsylvania) was formulated in a CMC gel. Briefly, AMP cells isolated from human placenta were grown to confluence and the supernatant (ACCS) was harvested. Detailed analysis of the secreted growth factor and cytokine profile has previously been reported.^28^

### Animals

A total of 104 female 10-week-old Sprague-Dawely rats (Charles River, Cambridge, Massachusetts) were housed 2 per cage, in 12/12 hours’ light/dark cycles and were given food and water ad libitum. They were allowed a period of 3 days for acclimatization. The Harvard Medical Area standing committee on animals approved all animal procedures (Protocol no. 04794), and all institutional guidelines for the care and treatment of laboratory animals were followed.

### Experimental groups

Animals were randomly assigned to 2 different treatment groups: Saline (control) and ACCS. Each group was further divided into 5 subgroups according to postoperative survival time points: 1, 2, 4, 6, and 8 weeks. The 1-week subgroups (n = 6) were used exclusively for histological studies, while animals in the subgroups pertaining to the other 4 time points (n = 13-16) were either assigned to histological studies, or to mechanical testing. Out of those, some specimens (n = 5) were used for collagen studies. An additional group (n = 10) was euthanized at time point 0 to test baseline tendon repair strength.

### Surgical procedure

Rats were anesthetized using Ketamine (60 mg/kg IP; Putney, Inc, Portland, Maine) and Xylazine (10 mg/kg IP; VEDCO, Inc, St Joseph, Missouri) and were maintained using 1% to 2% isoflurane (Hospira Inc, Lake Forest, Illinois) and oxygen. The right hind limb was shaved, disinfected using successive alternate applications of 70% alcohol (prepared from Surgipath Reagent Alcohol 100%, Leica, Richmond, Illinois) and 10% povidone iodine (Purdue Products LP, Stamford, Connecticut) scrubs. A longitudinal incision was made 1-cm proximal to the calcaneal insertion and sharp dissection was performed to expose the Achilles tendon, which was transected at its midpoint. The midsubstance of both the proximal and distal ends of the tendon was injected with either 100 μL of 0.9% sodium chloride solution (Hospira Inc, Lake Forest, Illinois) or ACCS in 100 μL CMC gel. Ends were then approximated and repaired using the modified Kessler technique^32^^,^^33^ with 6-0 braided polyester sutures (Ethicon, Somerville, New Jersey). Skin was closed using 5-0 nylon monofilament sutures (Ethicon), wrapped in Petroleum gauze (KENDALL, Mansfield, Massachusetts) and casted using a quick-drying casting tape (Scotchcast Plus, 3M) from toes to abdomen, achieving a 3-point stability (ankle-knee-hip).

### Postoperative period

The animals were housed individually for a period of 1 week to allow immobilization of the right hind limb. Three doses of postoperative analgesia using buprenorphine hydrochloride (0.005 mg/kg SC, Reckitt Benckiser Healthcare, United Kingdom) were given immediately after surgery and in the course of the following 48 hours. Rats were closely monitored daily. Cast removals were performed 1 week postoperatively to allow for weight bearing. Rats were housed 2 per cage till their assigned euthanasia time, ranging from 1 to 8 weeks.

Euthanasia was performed using an overdose of isoflurane (10%). Achilles tendons were dissected free of the extraneous soft tissue and harvested together with the calcaneal bone and parts of the gastrocnemius and soleus muscle complex. The material testing specimens were immediately frozen at – 80°C while specimens for histology were dissected in a similar fashion without the calcaneus. Three contralateral uninjured tendons from each animal subgroup were harvested as controls.

### Mechanical testing

**Figure 1:**
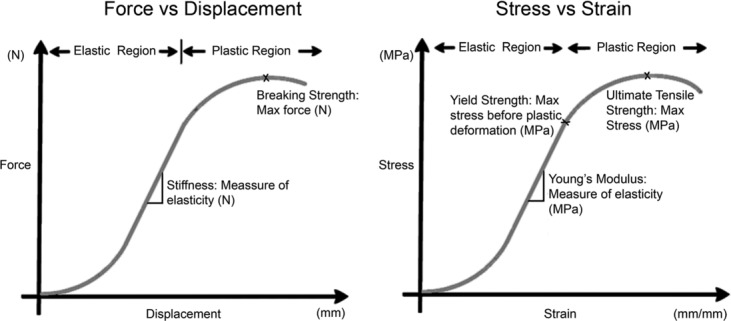
Examples of force versus displacement and stress versus strain curves. The results from mechanical testing of tendons were used to generate force versus displacement and stress versus strain curves, from which breaking strength, stiffness, yield strength, ultimate tensile strength, and Young's modulus could be extracted.

On the day of evaluation, specimens were thawed to room temperature and prepared for mechanical testing. The muscle was carefully separated from the proximal tendon by blunt dissection to produce a fan of tendon fibers^34^ that were then gripped by using a large Pennington clamp (Johnson & Johnson, New Brunswick, New Jersey). The distal end of the tendon was then gripped by using another Pennington clamp proximal to the calcaneal insertion. The 2 Pennington clamps were then vertically secured in an Instron 5565 material testing system (Instron, Norwood, Massachusetts) by using pneumatic grips with serrated jaw faces. Tendon width, radius, and length (distance between Pennington clamp ends) were recorded by using a digital Vernier caliper.^34^ The cross-sectional area was calculated, assuming circular tendon geometry. Tendons were kept moist by using gauze with saline throughout testing. Tendons were preconditioned for 3 cycles at 2% extension immediately followed by a load-to-failure test at a speed of 6 mm/min.^35^ Loading force was measured by using a 100-N load cell, and all data were collected with Blue-Hill 2 software (Instron). *Breaking strength* (defined as maximum force), *stiffness* (force required per unit displacement), *tensile strain* (defined as change in length over initial length mm/mm), *ultimate tensile strength* (defined as maximum stress or force per unit area), *yield strength* (defined as maximum stress in elastic region), and *Young modulus* (a measure of a material's resistance to elastic deformation) were obtained, and force versus displacement and stress versus strain curves generated (Fig 1).

### Histology

Six tendons from each treatment group at each time point were harvested for histology. These tendons were immersed in 10% formalin for 24 hours and then rinsed in phosphate buffered saline. Tendons were then cut longitudinally for histological evaluation. Paraffin-embedded sections were mounted onto slides and stained with H&E and Masson's Trichrome using standard protocols. Sixty tendons, representing different treatment groups at time points 2, 4, 6, and 8 weeks were used for tendon thickness measurements. An average of 3 transverse measurements taken at the midpoint, proximal, and distal ends were used to calculate tendon diameter and transverse cross-sectional area.

Fifteen longitudinal sections of rat Achilles tendon representing all treatment groups at 1, 2, 4, 6, and 8 weeks were examined “blindly,” not knowing the treatment or the time point for any tissue. Picrosirius red staining was utilized, which underpolarized light “stains” collagen type I yellow-orange and collagen type III green.

### Collagen studies

#### Chemicals

Pyridinoline standards were purchased from Quidel (San Diego, California) and pentosidine was purchased from Cayman Chemical Co. Hepatofluorobutyric acid was from Fluka (Sigma-Aldrich). Chloramine-T, perchloric acid, hydrochloric acid, and all other chemicals were purchased from Sigma (St Louis, Missouri) and were of analytical grade or better.

### Preparation of tissues

Following mechanical testing, rat tendons were sampled for hydration, total collagen content, and cross-link density from groups at 2, 4, 6, and 8 weeks. Hydration was determined by wet weight/dry weight measurements of lyophilized tissue. Approximately 2 to 5 mg samples were then hydrolyzed in 12M HCl in vacuo at 110°C for 18 to 24 hours. An aliquot of hydrolysate was taken for hydroxyproline analysis.

### Hydroxyproline assay

Hydroxyproline was measured using a 96-well microplate modification of a traditional colorimetric assay. Briefly, samples were diluted in assay buffer. Chloramine-T reagent was then added and allowed to react at room temperature for 20 minutes followed by the addition of dimethylaminobenzaldehyde reagent in perchloric acid. After heating at 60°C for 15 minutes, absorbance was read at 550 nm. Sample concentrations were then determined using a commercially available hydroxyproline standard. The values were used to determine collagen content assuming 14% hydroxyproline per collagen molecule. Collagen content was expressed as a percentage of dry weight.

### Collagen cross-link analysis with high-performance liquid chromatography

The mature trifunctional cross-links pyridinoline (hydroxylysylpyridinoline) and deoxypyridinoline (lysylpyridinoline) as well as the nonreducible, difunctional, glycation-associated, nonenzymatic cross-link pentosidine were measured on the basis of native fluorescence using an Agilent 1100 high-performance liquid chromatography system with diode array detector and fluorescence detector using Chemstation software (Rev A.10.02). The chromatographic method consisted of an initial 20-minute isocratic run of 20 mM heptofluorobutyric acid as an ion-pairing agent with 20% methanol (MeOH) followed by a 20% to 40% MeOH gradient over the next 20 minutes. Pump flow was 1 mL/min and column temperature 25°C. The fluorescence detector was programmed to switch from pyridinoline fluorescence (ex295/em395) to pentosidine fluorescence (ex328/em385) at 25 minutes to match the retention times of the different cross-links. Commercially available standards were used for cross-link quantitation and were expressed on a mole per mole basis with collagen (MW ˜300 kD). No prefractionation was necessary as the cross-link levels in the rat tendon samples were found to be sufficient in preliminary analyses.

### Statistical analysis

Data were subjected to 2-way analysis of variance, with treatment and time as independent factors. A Bonferroni post hoc test was used to determine statistical significance. *P* < .05 was considered statistically significant. All values are given as mean ± SD.

## RESULTS

### Material properties

A total of 104 rat Achilles tendons were transected, injected with saline or ACCS in CMC, then sutured, and allowed to heal for 0, 1, 2, 4, 6, or 8 weeks before being harvested. Overall, the surgical procedure was well tolerated, with a total of 3 rats (˜3%) lost postoperatively.

#### Breaking strength

**Figure 2:**
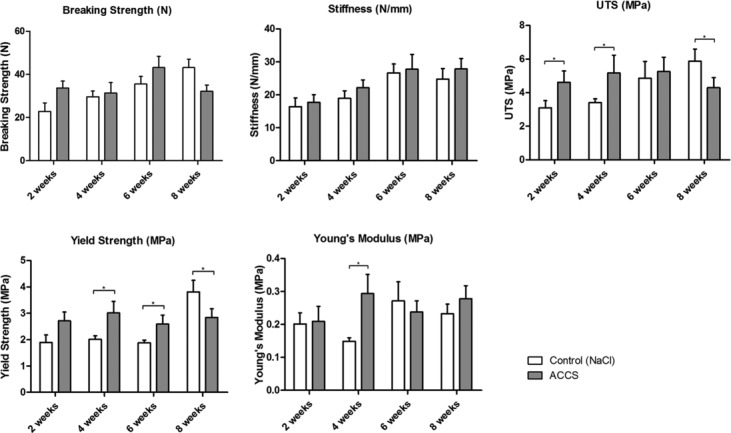
Material properties of tendons. Graphs summarizing the material properties of tendons treated with amnion-derived cellular cytokine solution (ACCS) and saline control 2, 4, 6, and 8 weeks after repair. UTS indicates ultimate tensile strength. ^a^*P* > .05.

At the 2-week time point, the ACCS in CMC-treated tendons showed significantly higher breaking strength compared to controls (Fig 2). Only at the 8-week time point, the controls demonstrate higher breaking strength than the ACCS in CMC group.

#### Stiffness

The tendons’ Stiffness increased over time but showed no significant differences between the study groups (Fig 2).

#### Ultimate tensile strength

The ACCS in CMC-treated tendons showed significantly higher ultimate tensile strength at weeks 2 and 4 compared to the saline-treated controls (Fig 2). At week 6, there was no difference between study groups observed, whereas at week 8, the saline-treated tendons showed significantly higher ultimate tensile strength.

#### Yield strength

The yield strength was higher in ACCS in CMC-treated tendons at weeks 2, 4, and 6, the 4- and 6-week time points being of statistical significance (Fig 2). At week 8, the saline-treated tendons showed significantly higher yield strength.

#### Young's modulus

The ACCS-treated tendons demonstrated a significantly higher Young's modulus at week 4 than saline-treated tendons (Fig 2). Uninjured tendons were found to have a Young's modulus of 240 Pa.

### Histology and immunohistochemistry

The lesions presented in all sections by bright field examination consisted of an irregularly linear array of immature connective tissue in either the repair phase or the remodeling phase, or perhaps both in different areas of individual sections. The slides were examined for the proportion of collagen type I versus collagen type III. Because of the variation encountered and subjective nature of this evaluation, samples were assigned to 1 of 3 groups:

1. More than 66% of the collagen (in the repair or remodeling zone) was type III
2. With variation, there was significant amounts of both collagen type III and collagen type I in the repair/remodeling zone.
3. Less than 33% of the collagen in the repair/remodeling zone was type III.

The results are presented in Table 1.

### Collagen studies

**Figure 3:**
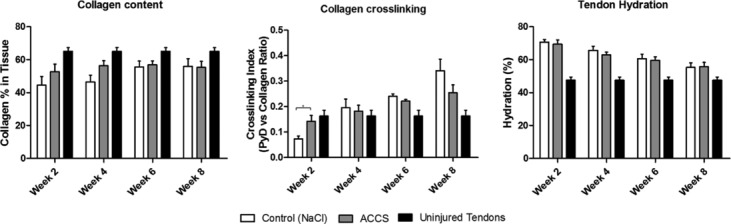
Graphs summarizing tendon collagen content, cross-linking, and hydration. A hydroxyproline assay was used to determine the collagen content in the tendons, assuming 14% hydroxyproline per collagen molecule. Collagen cross-link analysis was performed using native fluorescence and high-performance liquid chromatography. Tendon hydration was determined by wet weight/dry weight measurements of lyophilized tissue. ACCS indicates amnion-derived cellular cytokine solution. *P > .05.

As shown in Figure 3, tissue hydration was shown to decrease from approximately 70% to 55% over the course of 8 weeks in all groups, with no significant differences observed between groups.

It is well known that total collagen content rises rapidly during the “proliferative phase” (day 4-14) and levels stabilize at 4 to 5 weeks during the early portion of the “maturation phase.” As shown in Figure 3, by 2 weeks postinjury, total collagen content levels were 80% and 93% of the levels observed at 6 weeks for controls and ACCS in CMC, respectively, reflecting the fact that much of the collagen deposited into the healing wound bed has already occurred at 2 weeks. As compared to controls, the rise in content occurred faster in the ACCS in CMC group, with near maximal levels noted at 2 and 4 weeks. In this regard and in comparison to controls, the collagen content in the ACCS in CMC group showed an 18% (2 weeks) and 21% (4 weeks) increase.

Regarding cross-link density, at 2 weeks, there was a statistically significant increase for the ACCS in CMC group over controls (*P* < .05) (Fig 3). A smaller difference was seen for ACCS in CMC over controls at 4 and 6 weeks. By 8 weeks, the ACCS in CMC group had stabilized and levels were noted to be lower than in controls. Almost identical levels of pyridinoline were observed in both groups at 6 weeks. Pentosidine was not detected in any of the samples tested.

## DISCUSSION

Earlier studies showed that the treatment of transected tendons with AMP cells can positively effect the mechanical properties of healing tendons.^36^ In this study, we examined the biomechanical properties of rats Achilles tendons after transection and application of ACCS in a sustained-release formulation. The results obtained demonstrate significant beneficial effects of ACCS in CMC at early time points of 2 and 4 weeks.

*Breaking strength* is defined as the ability of a material to resist tension force to the point where it ruptures or breaks. The ACCS in CMC-treated tendons showed a significantly higher breaking strength than controls after 2 weeks. In the previous study by Philip et al, no direct effect on breaking strength in the ACCS in saline group was demonstrated, but it was shown that more tendons in the ACCS group had healed to the point where they could be mechanically tested, suggesting an earlier improvement in healing than in the saline group.

The *ultimate tensile strength* is the maximum stress that a material can withstand while being stretched or pulled before failing or breaking. In this study, ACCS in CMC significantly enhanced the tendons ultimate tensile strength at week 2 and 4 but not thereafter.

*Yield strength* is defined as the maximum stress a material can sustain before beginning to deform plastically. A trend toward improved yield strength in ACCS-treated tendons was observed from week 2 and onward and this trend became statistically significant at weeks 4 and 6.

The Young's modulus measures the relative stiffness of a material. Excessive or too little stiffness compared the one of a healthy tendon is not desirable. The ACCS-treated tendons demonstrated a significantly higher Young's modulus at week 4 compared to tendons treated with saline.

The higher collagen content and the significantly higher cross-link density of ACCS-treated tendons at early time points compared to the control may provide an explanation for the improvement of mechanical properties. In previous studies, trifunctional pyridinoline cross-links peaked at 4 to 6 weeks postwounding.^37^ Thereafter, the levels were variable and slowly decline over the course of 6 months. Amnion-derived cellular cytokine solution may have had a stabilizing effect on the healing wound, helping to advance the wound into a more long-term remodeling phase at an earlier time.

This study has certain limitations. The retention volume and cytokine release pattern was not studied in this experimental setup. However, in our previous study with ACCS in saline as treatment group, the lack of significant positive effects on tendon healing was anticipated to be caused by the apparent effects of enzymatic degradation. The positive results from the group with ACCS in CMC in this study suggest a sustained release of the cytokines. Carboxymethyl cellulose has been reported as a carrier for sustained drug release in the literature.^38^^-^40 In this context, a limitation of the study is that there is a saline control as negative control but no CMC-only group as vehicle control.

The improved mechanical properties of ACCS in CMC-treated rat tendons at early time points were demonstrated in this study. Significant enhancements were observed in breaking strength, ultimate tensile strength, yield strength, and Young's modulus. This could allow for higher loading at an earlier time, which has been shown to improve healing outcomes. The lack of positive effects of ACCS in CMC at 8 weeks might be due to the one time only application in the sustained-release media CMC. However, the early stages of tendon healing are the critical time points for patients with tendon injuries.

Future studies should investigate if multiple applications of ACCS in CMC prolong the positive effects on tendon healing.

## Figures and Tables

**Table 1:** Collagen I versus III content in tendons treated with ACCS or saline

Time point, wk	Treatment	Type I collagen, %	Type III collagen, %
1	ACCS	<33	>66
	Saline	<33	>66
2	ACCS	40-60	40-60
	Saline	40-60	40-60
4	ACCS	40-60	40-60
	Saline	40-60	40-60
6	ACCS	40-60	40-60
	Saline	>66	<33
8	ACCS	>66	<33
	Saline	>66	<33

ACCS indicates amnion-derived cellular cytokine solution.
